# An Investigation to Study Morphology, Nanomechanical Attributes, Transcriptomics, and Proteomics of Tumor Associated Macrophages Derived Extracellular Vesicles in the Tumor Microenvironment

**DOI:** 10.30683/1927-7229.2026.15.04

**Published:** 2026-05-12

**Authors:** T.K. Kulkarni, A. Banerjee, N. Banerjee, J. Cuffee, S. Newell, E. Armstrong, S.N. Deloatch, J. Jong Park, A.H. El-Hashash, S. Bhattacharya, H. Banerjee

**Affiliations:** 1 Mayo Clinic, Jacksonville, FL; 2 Boston Medical School, Boston, MA; 3 Moffit Cancer Center, Tampa, FL; 4 Elizabeth City State University Campus of The University of North Carolina, Elizabeth City, NC, USA

**Keywords:** Cancer, Tumor-associated macrophages, exosomes, Atomic Force Microscopy, cell morphology

## Abstract

**Introduction:**

This study investigates the molecular and biophysical changes in Tumor-Associated Macrophage (TAM)-derived exosomes (TAME) compared to exosomes from non-tumor microenvironment (TE) macrophages (MO-E). TAMs are immune cells infiltrating tumors that promote cancer progression.

**Materials and Methods:**

Characterizing the cargo of TAME via Next Generation RNA Sequencing protein microarray analysis and studying their morphological and nanomechanical properties using Atomic Force Microscopy (AFM).

**Results:**

Key Findings regarding Morphology showed TAME are significantly smaller (~50.6 nm) than MO-E (~64.8 nm).

TAME exhibit higher Young’s modulus (~15.5 MPa) indicating increased stiffness. Surface roughness ot TAME Is slightly higher (~3.64 nm) than MO-E (~3.51 nm). RNA sequencing revealed differential expression of genes involved in drug metabolism, cell cycle regulation, survival pathways, and immune modulation. Pathways such as NF-KB, PI3K/Akt, IL-13 signaling, and metabolic reprogramming are influenced by TAME cargo.

Proteomics studies showed several Top upregulated proteins include ORP150 (promotes VEGF secretion), CSRP1, C1qB, DNER, PEPD, with roles in tumor growth and metastasis.

Downregulated proteins include tumor suppressors like GOLPH2 and VNN1.

These protein profiles suggest TAME carry oncoproteins that support tumor proliferation, invasion, and immune evasion.

**Conclusion:**

This study demonstrates that TAM-derived exosomes are morphologically distinct and carry molecular cargo that modulates key cancer-related pathways, highlighting their potential as biomarkers and therapeutic targets. The significance lies in understanding TAME’s role in cancer progression, which could lead to novel diagnostic biomarkers and therapeutic targets, especially considering current limitations in cancer diagnosis accuracy.

## INTRODUCTION

A tumor is a heterogeneous mixture of host cells, secretions, and an ability to influence tissue of the host on a cellular, molecular, and physical level to promote tumor growth and progression. The tumor microenvironment (TME) is the cellular environment in which tumors exist, and the TME plays a significant role in the development and progression of cancer [1]. In the early development of tumor growth, a relationship between cancer cells and the TME is established to support cancer cell survival and promote invasion of surrounding tissue and cell metastasis. The TME has been linked with the formation of tumors, or tumorigenesis, due to the recruitment of tumor cells and surrounding cells. Other than influencing tumor growth, cancer development and progression, the TME is composed of cells and molecules that can promote angiogenesis, induce drug resistance, and suppress a host immune system [2].

The TME includes the surrounding cells such as immune cells, blood vessels, fibroblasts, signaling molecules and inflammatory cells. However, the components of the tumor microenvironment can vary between different tumor types. Within the TME there are interactions between malignant cells that invade healthy tissues and metastasize and nonmalignant cells that have protumorigenic (promoting tumorigenesis) functions in all stages of cancer formation by stimulating proliferation. Understanding the mechanisms and interactions that occur within the TME is important for cancer management and therapeutic strategies. Targeting the TME can help reduce drug resistance, improve treatment efficacy, and prevent cancer spreading [3,4].

Tumor-associated macrophages (TAMs, M2 phenotype) play a role in the formation of cancer as they are widely observed and investigated in various tumors. TAMs are usually the most abundant population within the TME due to their early leukocytes developed within the tumors. TAMs can be activated and polarized by tumor derived or associated molecules which promote tumor progression and metastasis. In previous studies, it has been confirmed that TAMs are present in tumor tissues at large amounts and help with tumor development. Due to the close relationship between TAMs and tumors, and their abundance in tumors, the number of TAMs in malignant tumors is associated with poor prognosis. In addition to tumor progression and metastasis, tumor-associated macrophages also promote cancer cell invasion and resistance to anticancer drugs [4–10]. Research has confirmed that TAMs secrete cytokines, which help stimulate the invasion, proliferation, and survival of cancer cells. Cytokines are produced from the recruitment of macrophages to the TME. It is hypothesized that recruitment of macrophages to become TAMs is dependent of certain conditions like hypoxia, inflammation and increase in lactic acid. However, TAMs are not categorized as a subtype of macrophages as they cannot be observed in a steady state. Tumor-associated macrophages have both the M1 and M2 phenotypes with the potential of repolarizing to M1 phenotype, but active TAMs have properties similar to the M2 phenotype [11–13].

Exosomes are extracellular vesicles that contain proteins, DNA and RNA. Exosomes are present in all bodily fluids, such as blood and cerebral spinal fluid (Srivastava *et al*., 2022). They can be easily isolated and purified. Exosomes possess traits for drug delivery as they do not catalyze a measurable immune response, their uptake by recipient cells occurs through various mechanisms, their surface can be functionalized for cell specific targeting, they are stable, and they can be lyophilized (freeze-dried) without impacting their structural or biological properties [14].

Recently, researchers have discovered that TAMs can release exosomes, which are small vesicles containing various biomolecules such as proteins, nucleic acids, and lipids. These TAM-derived exosomes (TAME) have emerged as important mediators of intercellular communication within the tumor microenvironment and beyond. TAME can suppress the anti-tumor immune response by various mechanisms. They can induce T-cell dysfunction and promote the expansion of regulatory T cells (Tregs), which inhibit effector T-cell function. Additionally, TAME may carry immune checkpoint molecules such as PD-L1, which can directly inhibit T-cell activity. TAME has been implicated in promoting tumor angiogenesis, the process by which new blood vessels form to supply nutrients and oxygen to the growing tumor. These exosomes can carry pro-angiogenic factors such as vascular endothelial growth factor (VEGF) and matrix metalloproteinases (MMPs), which stimulate endothelial cell proliferation and migration, enhance tumor cell invasion and metastasis by modifying the extracellular matrix and promoting epithelial-mesenchymal transition (EMT), a process that enables tumor cells to acquire invasive properties. These exosomes can transfer proteases and other molecules that facilitate tumor cell migration and invasion to distant sites [15,16].

Understanding the role of TAM-derived exosomes in cancer progression is important for the development of novel therapeutic strategies targeting the tumor microenvironment. Targeting exosome secretion or blocking specific exosome-mediated pathways could potentially help in inhibiting tumor growth, metastasis, and overcoming resistance to therapy, we performed comprehensive molecular characterization and biophysical analysis TAME and M0 macrophage derived exosomes (M0-E) by transcriptomic analysis using RNA Sequencing and Ingenuity Pathway Analysis (IPA) and Atomic Force Microscopy (AFM) respectively. We further investigated differential transcriptomic expressions, morphological attributes and nanomechanical properties of TAME and M0-E.

## MATERIALS AND METHODS

### Cell Culture

NCl-H209 (ATCC HTB-172), Human Monocyte THP-1 (ATCC TIB-202) were acquired from American Type Culture Collection (ATCC, Manassas, Virginia). Routine maintenance for each cell line was followed per ATCC protocol. All media were supplemented with 10% fetal bovine serum and 100 μg/mL penicillin/streptomycin. All cell lines were grown in 5% CO_2_ in 25 cm^2^ filter cap flasks and cultured in a laboratory incubator at 37°C.

### TAM Induction

THP-1 cells were differentiated into macrophage-like cells, M0 by incubation with phorbol 12-myristate 13-acetate (PMA) concentrations at 40 ng/mL after 24 hours incubation. In six-well plates, M0 macrophage cells were grown to confluence, placed over the macrophages were inserts with 0.4 μm micropores, which contained HTB172 lung cancer cells grown to confluence along with controls with no cancer cells. These cells were cultured for a period of 48 hours before exosomes were isolated.

### Exosome Isolation

Exosomes were isolated following the total exosome isolation kit purchased from Invitrogen, Carlsbad, CA (Catalog # 4478359). As per the manufacturer’s protocol, from a confluent flask of cells, culture media was collected in a 15 mL centrifuge tube and centrifuged at 14,000 rpm, 10 min. Supernatant was discarded and the pellet was re-suspended in 5 mL of appropriate incomplete media (no Fetal Bovine Serum), placed in a 25 cm^2^ culture flask, and allowed to grow for up to 12 hours. This was done to eliminate the high levels of exosomes contained in FBS and to prevent contamination of the cell derived exosomes. After growing cells in incomplete media for 10 hours, cells in media were collected into a 15 mL centrifuge tube and centrifuged at 14,000 rpm for 10 min. Supernatant (1 mL) was placed in each of 5Eppendorf tubes (1.5 mL). Supernatant was then centrifuged at 2,000 × g for 30 min to remove cells and debris and supernatant was then transferred from each tube and placed into a new 1.5 mL Eppendorf tube without disturbing the pellet (total 5 tubes). Total exosome isolation reagent (0.5 volumes) was added to each of the 5 tubes containing cell-free culture media. The culture media/reagent mixture was vortexed until a homogenous solution appeared, and the mixture was incubated overnight at 4°C. After incubation, the samples were centrifuged at 10,000 × g for 1 hour at 4°C in the benchtop centrifuge 5804 R (Eppendorf, Hauppauge, NY). The supernatant was aspirated and discarded leaving the pellet containing exosomes, which was not readily visible in most cases. The pellet was then resuspended in between 1 mL 1X PBS and stored in minus eighty-degree Celsius freezer.

### RNA Isolation

Total RNA was isolated using the Single Cell Real-Time RT-PCR Assay Kit Catalog # CL-0003 (Signosis Inc., Santa Clara, CA) following a standard protocol. Briefly, after resuspension buffer was added to the exosome pellet created using Total Exosome Isolation procedure detailed above, the sample was incubated for 5–10 min at room temperature to permit the pellet to dissolve followed by thorough resuspension. One volume of pre-warmed (37°C) 2X Denaturing Solution was added and mixed. In a 1.5 mL RNase-free Eppendorf tube, 1X PBS was added to the exosome sample so that the total volume was 200 μL. The mixture was incubated on ice for 5 min and one volume of Acid-Phenol: Chloroform was added. The sample was vortexed for 45 seconds before centrifuging at 10,000 × g for 5 min to separate the mixture into aqueous and organic phases. The aqueous (top) phase was removed and transferred to a fresh RNase-free tube and the organic (bottom) phase discarded. Ethanol (100%) was added as 1.25 vol to the aqueous phase and mixed thoroughly. A filter cartridge was placed into one of the collection tubes and 700 μL of the lysate/ethanol mixture was pipetted onto the filter cartridge. Sample was centrifuged at 10,000 × g for approximately 15 s, until the mixture passed through the filter. The flow-through was discarded and the centrifuge process was repeated until all the mixture passed through the filter. The collection tube was saved for washing steps. RNA Wash Solution 1 (700 μL) was added to the filter cartridge and pulse centrifuged at 10,000 × g. Flow-through was discarded and new filter cartridge was placed into the same collection tube. Wash Solution 2 (500 μL) was added to the filter cartridge and pulse centrifuged at 10,000 × g. Flow through was discarded. An additional 500 μL of Wash Solution 2 was added to the filter. After discarding the flow-through from the last wash, the filter cartridge was placed into the same collection and centrifuged for 1 min to remove any residual fluid from the filter. The filter cartridge was transferred to a fresh collection tube and 50 μL of pre-warmed (95°C) elution Solution or nuclease-free water was applied to the center of the filter. Sample was centrifuged for 30 s to recover RNA. Elution was repeated once more with an additional 50 μL of Elution Solution of nuclease-free water. The RNA (eluate) was collected and placed on ice for immediate use or stored at ≤ −20°C. RNA concentration was determined using a BioMate 3 Spectrophotometer (Thermo Fischer Scientific Inc., Waltham, MA spectrophotometry.

### RNA Sequencing and Ingenuity Pathway Analysis (IPA)

The RNA exosome sequences were generated from Prim Bio Research Institute (Exton, PA) a contract vendor for Next Generation Sequencing. Ingenuity Pathway Analysis (IPA) Licensed from Qiagen, CA, USA was used to organize and analyze data obtained from the RNA sequences of exosomes. Differentially expressed genes creating the core canonical pathways and their importance and role in the Tumor Microenvironment (TME) were determined by using the Gene Ontology and KEGG (Kyoto Encyclopedia of Genes and Genomes) biological pathways related to differentially expressed genes in TAM versus non-TAM exosomes.

### AFM Experimental Methodology

Freshly cleaved mica, known for its atomically smooth surface and slight negative charge, is frequently selected as a substrate for nanoscale biological imaging. In this study, mica served as the platform for evaluating how different treatments influence exosome’s morphology and mechanical behavior. To promote firm attachment of exosomes, the mica surface was functionalized prior to use. Specifically, a 3:1 (v/v) mixture of 3-aminopropyltriethoxysilane (APTES) and N,N-diisopropylethylamine (DIPEA) was applied to newly cleaved mica and incubated for 2 hours at 60 °C. This silanization renders the surface positively charged, allowing efficient binding of the naturally negatively charged exosome membranes. The modified mica was used immediately. For AFM analysis, exosome suspensions were diluted 1:1000 in Milli-Q water. Milli-Q water was chosen instead of PBS to prevent crystallized salt residues, which can distort topography and interfere with mechanical measurements. A 5 μL aliquot of this diluted sample was deposited onto the functionalized mica and allowed to adsorb for approximately 30 minutes. After incubation, the surface was gently rinsed with Milli-Q water to remove excess, unbound vesicles.

Atomic force microscopy was performed on a Dimension Icon ScanAsyst system (Bruker, Santa Barbara, CA). Exosome morphology and nanomechanical parameters were quantified using two complementary approaches: Peak Force Quantitative Nanomechanical Mapping (PF-QNM) and nanoindentation. All measurements were conducted in a liquid environment to preserve native vesicle structure. PF-QNM, a peak-force–based tapping mode, intermittently contacts the sample while maintaining controlled interaction forces, thereby generating high-resolution height maps along-side simultaneous mechanical property measurements.

A ScanAsyst-Air probe equipped with a ~5 nm–radius pyramidal tip and a cantilever of nominal spring constant 0.4 N/m was utilized to obtain high-resolution exosome topography. Prior to imaging, the laser was carefully positioned on the cantilever’s gold-coated backside to maximize signal quality. Topographical scans were collected using a gentle peak force of 300 pN and a slow scan rate of 0.1 Hz. A minimum of fifty vesicles were imaged to ensure robust statistical evaluation of surface features. Height and roughness measurements were extracted using Nanoscope Analysis v1.9. Exosome height values were obtained through the software’s sectional analysis tool, while surface roughness (reported as RMS height variation) was quantified by selecting only the vesicle region, taking care to exclude surrounding background artifacts.

For nanomechanical characterization, the AFM probe was calibrated in Milli-Q water on freshly cleaved mica to determine its actual spring constant (0.38 N/m) and deflection sensitivity (35 nm/V). Performing this calibration in a liquid environment is critical to account for hydrodynamic drag effects typically encountered during fluid-phase AFM experiments. Nanoindentation measurements were carried out on at least 50 exosomes, with each indentation producing a force–separation (F–S) curve. The probe was brought into contact with the sample using a trigger force of 600 pN. Each F–S curve was subsequently analyzed using the Derjaguin–Müller–Toporov (DMT) model to extract nanomechanical parameters including Young’s modulus, deformation, and adhesion. The DMT model was appropriate given the modest indentation depth relative to exosome size (tens of nanometers) and the measurable adhesive interactions at the tip–sample interface.

### Proteomic Studies

Combining direct antigen-labeling technology with a vast library of array-validated antibodies, RayBiotech LLC, USA has created the largest commercially available antibody array to date. With the L-Series high density array platform, researchers can now detect thousands of proteins simultaneously, obtaining a broad, panoramic view of protein expression. The first step in using the RayBio® L-Series Antibody Array is to biotinylate the primary amine groups of the proteins in the sample, TAME and non-TAME cell lysates, The glass slide arrays are then blocked by a standard blocking buffer, and the biotin-labeled sample is then added onto the glass slide, which is pre-printed with capture antibodies. The slide is incubated to allow binding of target proteins. Streptavidin-conjugated fluorescent dye (Cy3 equivalent) is then applied to the array. Finally, the glass slide is dried, and laser fluorescence scanning is used to visualize the signals.

## RESULTS

### TAME Transcriptomic Data Analysis by the Ingenuity Software

**Figure 1:**
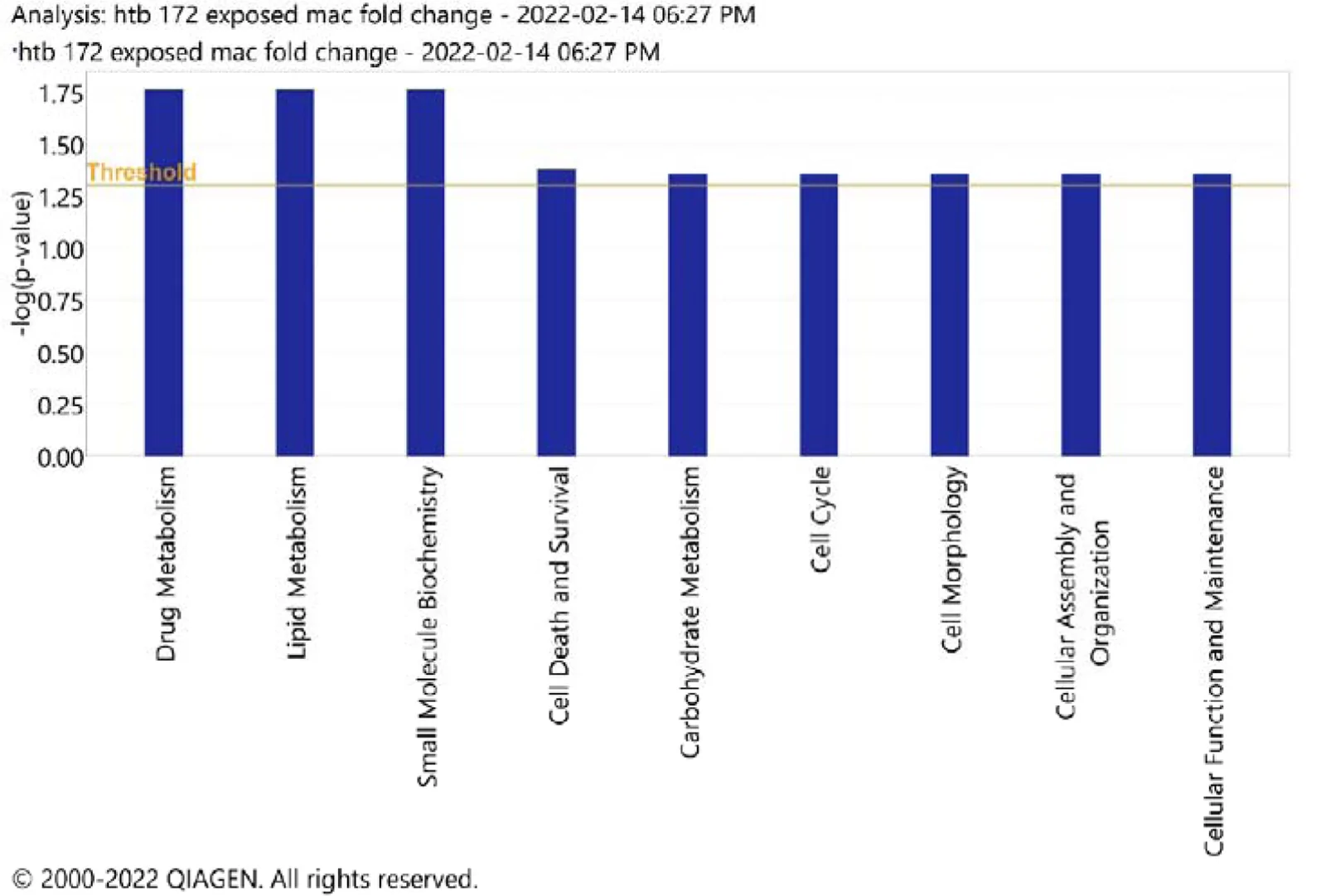
IPA analysis of TAME Transcriptomic cargo as obtained by RNA Sequencing showing different cell signaling pathways that could be influenced on target cells in the TME.

**Figure 2:**
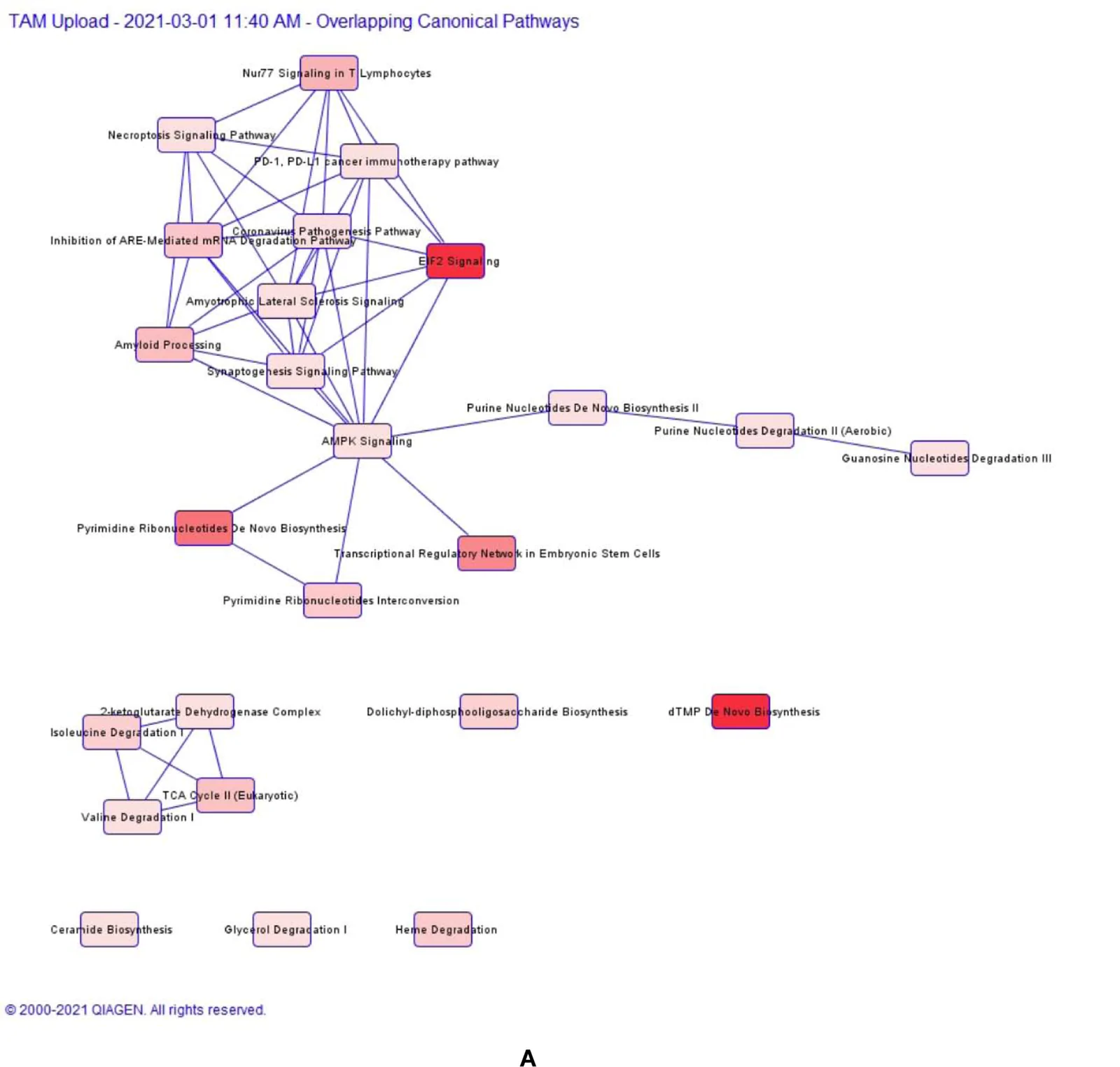

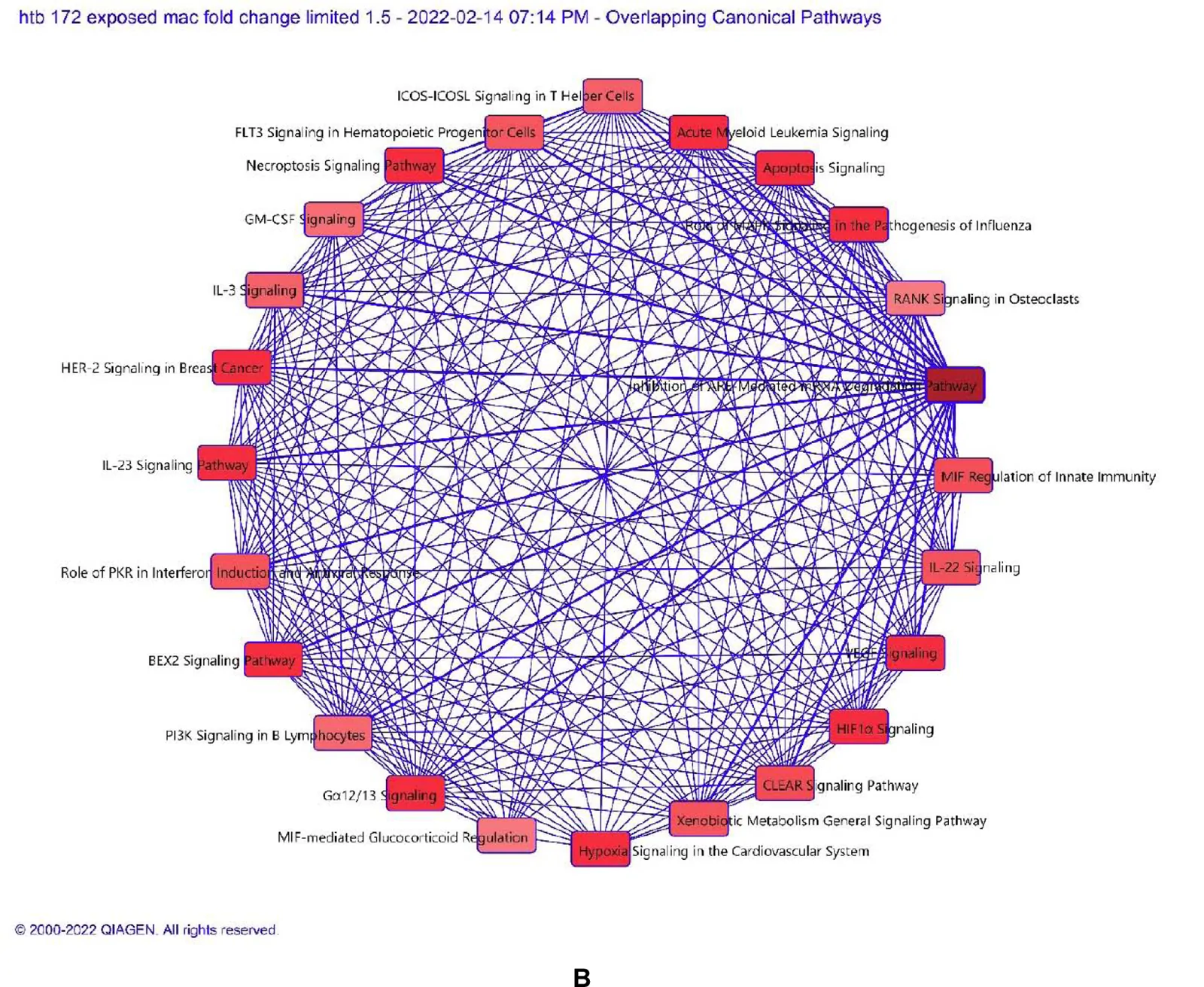
**A**: Core Canonical pathways and genes that could be influenced by the TAME transcriptomic cargo in target cells in the TME as obtained by IPA. **B.** Synopsis of overlapping Canonical pathways and genes that could be influenced by the TAME transcriptomic cargo in target cells in the TME as obtained by IPA.

The TAME transcriptomic data analysis by the Ingenuity Software showed several pathways to be involved that could be modulated by the RNA cargo of the TAME (Figure 1). Additional IPA pathway analysis showed that there is a definite interaction between several core canonical pathways that could be influenced by the TAME RNA load (Figure 2A and 2B).

Surface roughness of TAME is slightly higher (~3.64 nm) than M0-E (~3.51 nm). RNA sequencing revealed differential expressions of genes involved in drug metabolism, cell cycle regulation, survival pathways, and immune modulation. Pathways such as NF-κB, PI3K/Akt, IL-13 signaling, and metabolic reprogramming are influenced by TAME cargo.

### Evaluating the Morphology and Nanomechanical Attributes for Macrophage-Derived Exosomes

**Figure 3:**
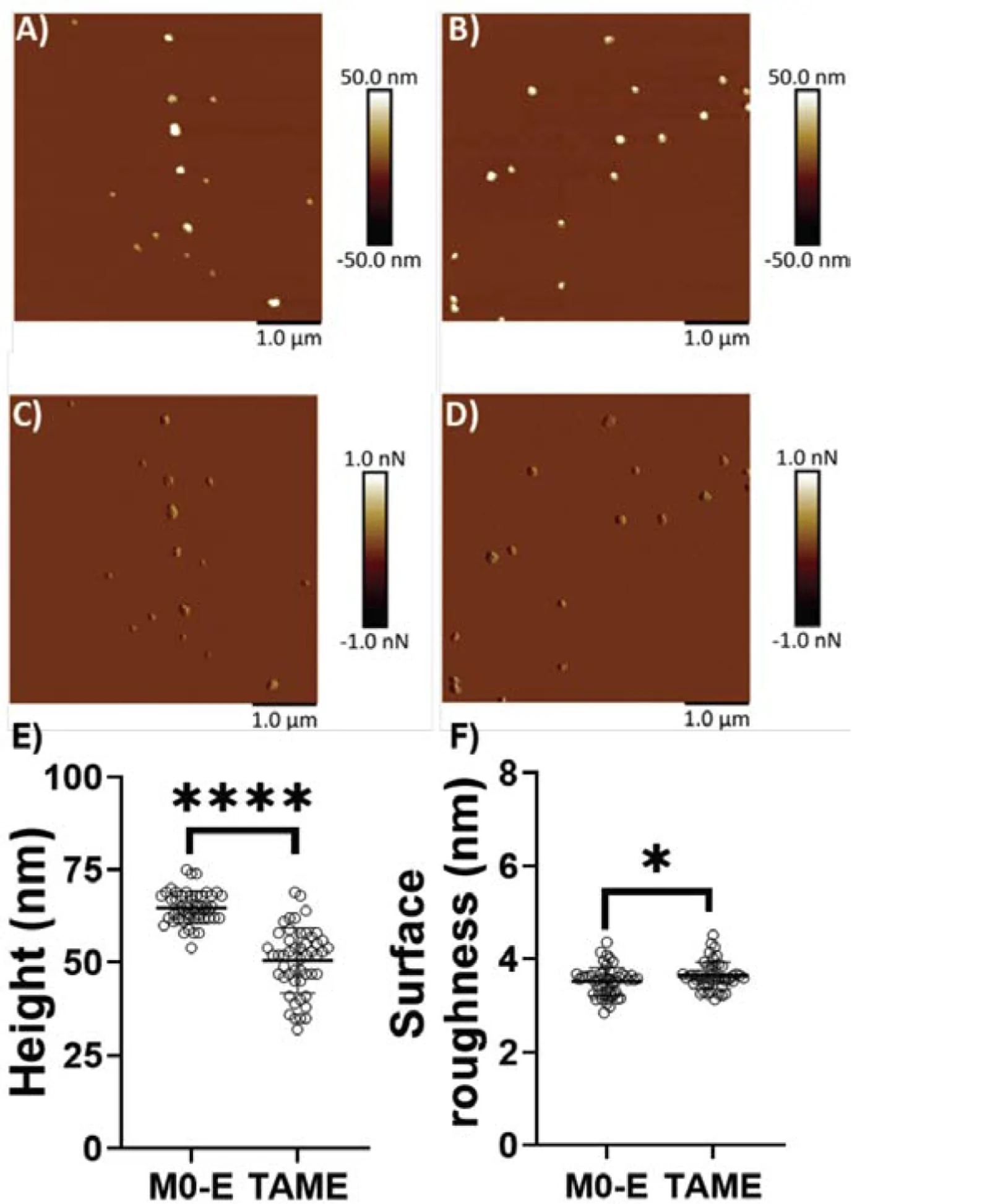
Representative surface topography of exosomes under various conditions. **A**) Height image and **C**) Peak force error image of M0 derived exosomes (M0-E). **B**) Height image and **D**) Peak force error image of TAM derived exosomes (TAME). Morphology quantifications. **E**) Height. **F**) Surface roughness. (Statistical significance performed by One Way ANNOVA: *, p<0.05, ****, p<0.0001)

We evaluated and compared the morphology and nanomechanical attributes for TAME and M0-E. Mica surface is used as a base substrate to adsorb exosome sample. We identified the samples on mica surface using AFM’s PF-QNM technique and quantified them in terms of their height and surface roughness. Height images are used to accomplish the quantification. We also displayed peak force error images of the exosomes under various conditions to improve their qualitative value. We observed no significant alteration in mica surface topography after APTES: DIPEA treatment (data not shown). Representative height profile and peak force error images for M0-E are displayed in Figure 3A–3B, respectively. We also exhibit morphology of TAME in Figure 3C–3D in terms of its height profile and peak force error, respectively. These studies were performed on three exclusive occasions and additional relevant data pertaining to the same is displayed in supplementary Figure S1. Using NanoScope Analysis software, we quantified the height and the surface roughness for both TAME and M0-Es. The average height of M0-E was 64.82±4.28 nm that was significantly bulky compared to TAME which was recoded as 50.6±8.72 nm (Figure 3E). Interestingly, the average surface roughness for M0-E exhibited 3.51±0.31 nm, which is slightly less than their TAM counterparts displaying an average surface roughness of 3.64±0.28 nm (Figure 3F).

**Figure 4:**
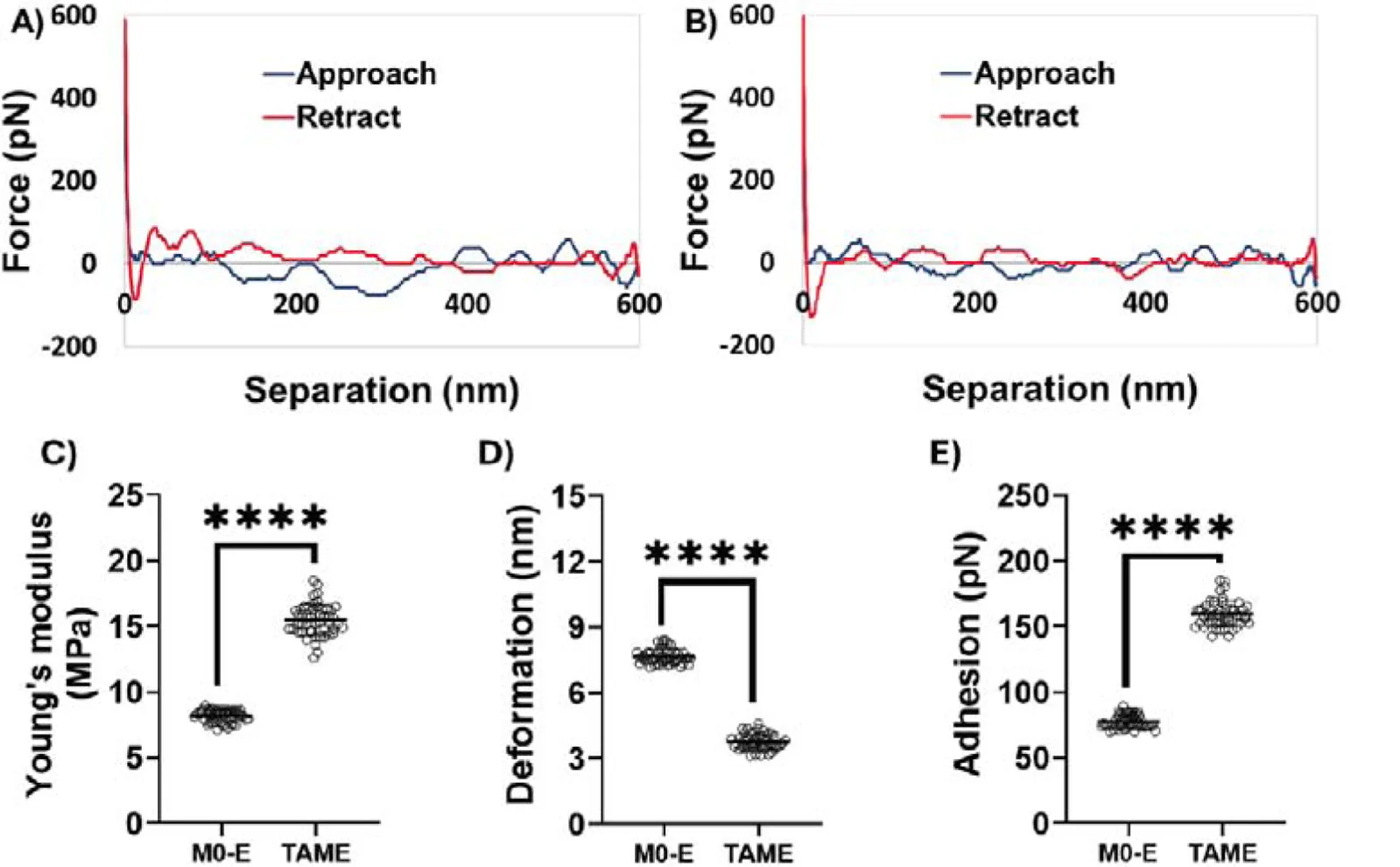
Nanomechanical characterization of macrophage derived exosomes under various treatments. A representative force-separation curve for exosomes derived from **A**) M0 macrophage and **B**) TAM. Nanomechanical attributes **C**) Young’s modulus. **D**) Deformation. **E**) Adhesion. (Statistical significance performed by One Way ANNOVA: ****, p<0.0001).

Nanomechanical attributes are evaluated using the exact same ramping parameters to maintain consistency in their values. A representative F-S curve from TME non-exposed and exposed exosomes derived from macrophages is shown in Figure 4A–4B, respectively. Flat baseline indicates a complete cycle of traction and retraction between the AFM tip and the exosome sample surface. Only such F-S curves are employed for analysis purposes using DMT contact mechanics’ model. As shown in Figure 4C, M0-Es were significantly softer than their counterparts. Notably, the Young’s modulus for M0-E and TAME were observed to be 8.19±0.45 MPa and 15.5±1.19 MPa, respectively.

Deformation on the other hand, displayed a complementary trend in its values compared to the Young’s modulus. Thus, deformation for M0-E was observed to be 7.65±0.32 nm and that for TAME to be 3.75±0.34 nm (Figure 4D).

Finally, the adhesion parameter for M0-Es observed to be 77.94±4.93 pN, which was significantly lesser than their counterparts that showed adhesion parameter of 159.47±9.69 pN (Figure 4E). Such trend in adhesion and Young’s modulus could indicate that for a constant applied force, stiffer exosome samples exert more adhesive pull on the AFM tip prior to its complete retraction, compared to the softer exosome samples. Such characteristic attributes show promise in detecting and differentiating macrophage-derived exosomes exposed to various treatments.

### Proteomic Data Analysis

The differentially expressed proteins in the TAME and non-TAME Macrophage cell lysates are represented in Tables 2 and 3. Only top ten highly expressed proteins and top ten most lowly expressed proteins have been described in this manuscript along with their significance in the Tumor Microenvironment in the discussion section. Proteomics studies showed several Top upregulated proteins include ORP150 (promotes VEGF secretion), CSRP1, C1qB, DNER, PEPD, with roles in tumor growth and metastasis. Downregulated proteins include tumor suppressors like GOLPH2 and VNN1.

These protein profiles suggest TAME carry oncoproteins that support tumor proliferation, invasion, and immune evasion.

## DISCUSSION

Tumor-associated macrophages (TAMs) are immune cells that infiltrate the tumor microenvironment and play critical roles in cancer progression and metastasis. Recently, researchers have discovered that TAMs can release exosomes, which are small vesicles containing various biomolecules such as proteins, nucleic acids, and lipids. These TAM-derived exosomes have emerged as important mediators of intercellular communication within the tumor microenvironment and beyond. There are several cancer-related core canonical pathways that are influenced by TAM derived exosomes, to study the role of TAM derived exosomes in lung cancer TME, we analyzed by RNA Sequencing and IPA analysis of the TAM exosomal RNA load. Here we describe the TME related cellular pathways as identified by our findings that we could attribute to tumor-associated macrophage-derived exosomes as described in current literature.

Our findings showed involvement of TAME Transcriptome in drug metabolism pathway, in terms of drug metabolism, TAMs can impact the efficacy of cancer therapies through several mechanisms: TAMs undergo metabolic reprogramming in the tumor microenvironment, which can influence their function and the surrounding cells. This metabolic adaptation can alter the availability of metabolites necessary for drug metabolism and affect the response to therapy. TAMs express various drug metabolism enzymes, including cytochrome P450 enzymes, which are involved in the metabolism of many drugs. The expression levels and activities of these enzymes in TAMs can influence the metabolism and efficacy of anti-cancer drugs. TAMs secrete chemokines and cytokines that can modulate drug metabolism in neighboring cancer cells. These factors can alter the expression of drug-metabolizing enzymes or transporters in cancer cells, impacting drug efficacy and resistance [17].

We found TAME transcriptome could influence cell cycle and cell survival. TAMs secrete various cytokines and growth factors that can influence cell cycle and cell survival. For instance, TAM-derived factors such as vascular endothelial growth factor (VEGF), transforming growth factor-beta (TGF-β), and interleukin-10 (IL-10) can promote tumor cell survival by stimulating angiogenesis, suppressing immune responses, and enhancing anti-apoptotic pathways. TAMs can also promote tumor cell survival indirectly by promoting the formation of new blood vessels (angiogenesis) within the tumor microenvironment. This increased blood supply ensures a steady delivery of oxygen and nutrients to tumor cells, thereby supporting their survival and proliferation. TAMs can facilitate tumor cell survival by remodeling the extracellular matrix (ECM) within the tumor microenvironment. This remodeling can promote tumor cell invasion and metastasis while also providing structural support to tumor cells, thereby enhancing their survival in the harsh tumor environment. TAMs can influence tumor cell survival by inducing metabolic changes within the tumor microenvironment. For example, TAMs can promote aerobic glycolysis (the Warburg effect) in tumor cells by providing them with lactate and other metabolic intermediates, thereby supporting their energy demands and survival [18].

The BEX2 gene, also known as Brain Expressed X-linked 2, has been implicated in various cellular processes, including cell cycle regulation, apoptosis, and differentiation. Its role in cancer and specifically in the signaling pathways related to TAMs is an area of active research. Several studies have suggested that BEX2 may be involved in tumor progression and metastasis by regulating processes such as epithelial-mesenchymal transition (EMT) and metastasis-associated phenotypes. However, the specific signaling pathways through which BEX2 influences TAMs and tumor progression are not yet fully elucidated and likely depend on the context of the tumor microenvironment and the specific cancer type. Some potential signaling pathways that may be involved in the interplay between BEX2 and TAMs include NF-κB signaling pathway that is a key regulator of inflammation and immune responses. TAMs can influence NF-κB activation, and BEX2 may modulate this pathway to affect tumor progression [19].

IL-13 signaling can influence the polarization of macrophages towards the M2-like phenotype, promoting their pro-tumor functions. The interaction between TAMs and the IL-13 pathway underscores the complexity of the tumor microenvironment and the multiple ways in which immune cells and cytokines contribute to cancer progression. Strategies targeting TAMs or IL-13 signaling are being explored as potential therapeutic approaches in cancer treatment, aiming to modulate the immune response and hinder tumor growth and metastasis. TAMs can influence the PI3K pathway within the tumor microenvironment through various mechanisms. For example, secretion of growth factors and cytokines by TAMs, such as EGF (Epidermal Growth Factor), PDGF (Platelet-Derived Growth Factor), and CSF-1 (Colony-Stimulating Factor 1) can activate RTKs on tumor cells, leading to PI3K pathway activation. TAM-secreted factors can also activate PI3K signaling in TAMs themselves, promoting their survival, polarization toward the M2-like phenotype, and pro-tumoral functions. TAMs can directly interact with tumor cells, leading to the activation of signaling pathways, including PI3K/Akt, which promotes tumor cell survival, proliferation, and invasion [20]. Therefore, the core canonical pathways derived by IPA analysis of the TAM derived exosomal transcriptome has a definite role in promoting cancer cells growth and survival in the Tumor Microenvironment. Notably, recent studies have suggested that TAMs-derived exosomes play crucial roles in malignant cell proliferation, invasion, metastasis, angiogenesis, immune responses, drug resistance, and tumor metabolic reprogramming. TAMs-derived exosomes have the potential to be targeted for tumor therapy.

Our AFM data on TAME is also significant as we have shown for the very first time that there is a distinct difference in morphology of TAME than exosome morphology derived from healthy M0 macrophages. This could be a very important finding as a new mechanism of cancer biomarker discovery. Diagnosing cancer is still dependent on the eye of the pathologist and false negative and false positive cancer diagnosis from biopsy samples is very common. This can lead to increased mortality and morbidity of the patient population and, therefore, further exploration of the TAM related exosomal morphology by AFM could help both the cancer research community, oncologists and pathologists for early detection of cancer, preventive and therapeutic strategies.

During our Proteomic analysis experiments, the top ten proteins that were found to be upregulated and downregulated for TAME in comparison non-TAME via difference in protein expression and fold change calculation. The roles of these proteins have been researched in various cancers, but it is unknown in tumor-associated macrophages. The top ten proteins found to have the most significant signal intensity for upregulation in M2 macrophages exposed to TME compared to M0 macrophages are ORP150, ALAD, CSRP1, C1qB, beta-1 Spectrin, DNER, eIf4a1, Col6A2, PEPD, and ADH proteins. They are oncoproteins in various cancers, except for ALAD. ORP150 has the greatest signal intensity in the data and is established as an oncoprotein due to promoting VEGF secretion, cell proliferation and reducing apoptosis in cancer cells (Rao *et al.,* 2021) CSRP1 is a protein that is found to be elevated in colon adenocarcinoma, prostate cancer, and acute myeloid leukemia (Yu *et al.,* 2023). C1qB promotes tumor growth in cervical cancer (Mangogna *et al.,* 2019), eI4a1 is highly expressed in prostate cancer (Xue *et al.,* 2021), and DNER is involved in the development in tumors, promotes EMT transition, proliferation, and metastasis within breast cancer cells (Wang *et al.,* 2020; Wang *et al.,* 2019). PEPD is established as an oncoprotein due to suppression and inhibition of tumor suppressor gene, p53, (Yang *et al.,* 2017) Col6A2 and ADH are associated with poor prognosis in glioma and hepatocellular carcinoma, respectively (Zhu *et al.,* 2022; Wu *et al.,* 2021). In comparison, upregulation of ALAD protein is a tumor suppressor within breast cancer cells and its downregulation is associated with poor prognosis (Ge *et al.,* 2017). No research has been published yet to check whether beta-1 Spectrin protein is an oncoprotein or tumor suppressor within cancer cells.

In this study, the top ten downregulated proteins were GOLPH2, SORD, PSMB6, FDPS, Amylin, MINPP1, Thrombospondin-1, PSMA3, LIMS1, and VNN1. Downregulated proteins found to be tumor suppressors were SORD, Amylin, Thrombospondin-1, and VNN1. SORD has been researched to promote inhibition of tumor growth in hepatocellular carcinoma (Lee *et al.,* 2022), while Amylin protein is activated in p53 deficient cancers that are capable of regression (Garnett *et al.,* 2021). Thrombospondin-1 is a cell cycle regulator that inhibits angiogenesis and expression levels are positively regulated by several tumor suppressor genes (Isenberg and Roberts, 2020). VNNI limits tumor growth by limiting the aerobic glycolysis pathway, a pathway that cancer cells rely on to meet high energy demands for growth and tumor maintenance (Giessner *et al.,* 2018). Compared to downregulated proteins established as tumor suppressors, GOLPH2, PSMB6, FDPS, MINPP1, PSMA3, and LIMS1 are oncoproteins. GOLPH2 overexpression is associated with cell proliferation and migration. *GOLPH2* knockdown leads to a decrease in proliferation and migration in lung cancer (Aruna and Li, 2018), while *PSMB6* knockdown is deadly to the survival of myeloma cells (Shi *et al.,* 2020). FDPS is an oncoprotein in prostate cancer and its downregulation can decrease cell growth and proliferation (Seshacharyulu *et al.,* 2019). MINPP1 inhibits the function of tumor suppressor, PTEN (Gimm *et al.,* 2001), while PSMA3 downregulation can suppresses tumor growth in gastric cancer (Yang *et al.,* 2022). LIMS1 promotes pancreatic cancer survival under oxygen-glucose deprived conditions (Huang *et al.,* 2019). Although current research has identified the roles of upregulated and downregulated proteins in various cancers, further research is still needed to determine the functional roles of these proteins as either oncogenes or tumor-suppressors in tumor-associated macrophages (TAMs).

Additionally, through transcriptome and proteomics analysis on macrophages exposed to cancer cells, we found a comprehensive list of genes and proteins that were observed to be altered compared to non-exposed macrophages. From the list of genes, we identified IL-12, β-tubulin, α-actin, cyclin A, B and D, focal adhesion kinase (FAK) among several others that have been shown to alter membrane dynamics in cells. Moreover, previous proteomics study recognized membrane surface proteins such as actin, FAK, spectrin TGFβ1 and NFκB from the comprehensive list; Cell membrane surface reflects various cellular processes and tends to alter its architecture resulting into changes in nanomechanical attributes such as stiffness, adhesion and deformation. The above-mentioned genes have been shown to alter membrane dynamics. For instance, IL-12 synthesized by Mesangial cells can stimulate cytoskeletal reorganization, while β -tubulin and α-actin maintain cellular integrity and alter during various cellular processes such as endocytosis and cell migration. The ruffling lamellipodia also contributes to overall alterations in membrane surface roughness in cells. Interestingly, FAK has been an accomplice to cell motility and regulates the flow of signals from the extracellular matrix to the actin cytoskeleton, causing its reorganization that leads to an alteration in membrane stiffness. Cyclins have been known to affect cell cycle phase, which involves reorganization of actin and mictrotubules to facilitate cellular transition from G0/G1 to G2/M phase.

Remarkably, TGFβ1 is a key activator that stimulates EMT and can induce cell migration and actin cytoskeletal reorganization, while NFκB causes depolymerization of microtubules thus altering the cytoskeletal reorganization. Cytoskeleton is responsible for transferring the cytoarchitecture alterations to gene transcription. In the current study, the exosomes are derived from macrophages exposed to cancer cells, and the alteration in cell membrane architecture could potentially extend to exosomes. Hence, we observe alterations in nanomechanical and morphological attributes in the exosomes derived from macrophages exposed to cancer cells. However, validation studies are still needed to confirm the role of these proteins and genes in exosomes, and this will be our future study.

Diagnosing cancer is still dependent on the eye of the Pathologist and false negative and false positive cancer diagnosis from biopsy samples is very common leading to increased mortality and morbidity of the patient population, henceforth, further exploration of the TAM related exosomal morphology by AFM could help both the cancer research community, oncologists and pathologists for early detection of cancer, preventive and therapeutic strategies.

## Supplementary Material

Supplementary Figure

SUPPLEMENTARY FIGURE

The supplementary figure can be downloaded from the journal website along with the article.

## Figures and Tables

**Figure 5:**
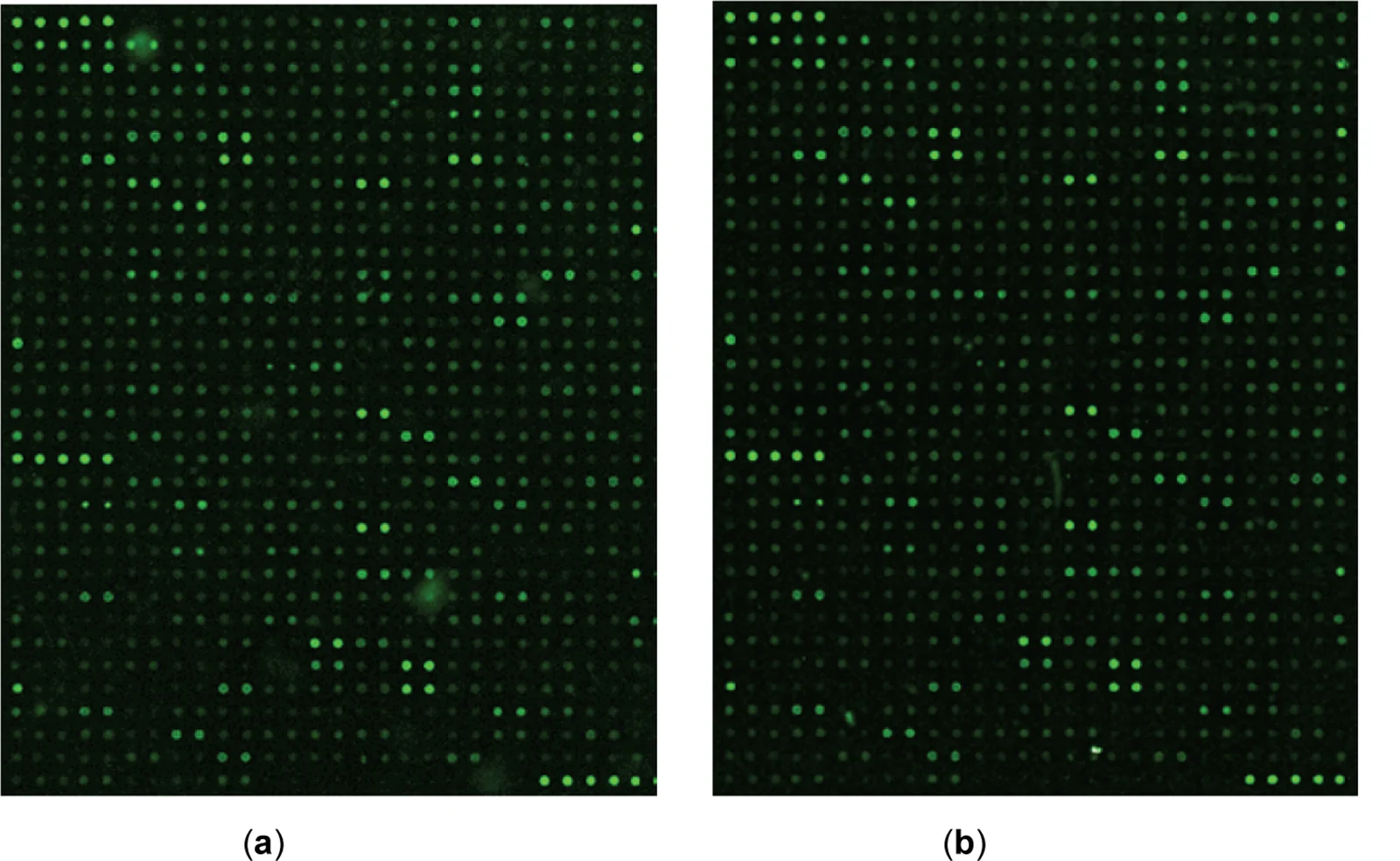
Protein array images of TAME derived cell lysates (**a**) in comparison with undifferentiated Macrophages Exosome lysates (**b**).

**Table 1:** Table Showing RNA Sequencing Result of Down Regulating Gene Expression, Several Gene Involved in Cellular Phagocytosis Showed Decreased Expression in TAM Compared to Unexposed Macrophages

Gene ID #	Gene Name	Fold Down Regulation	Name	Description
ENSG0000012422	MTUS1	0.018827587	Microtubule associated scaffold protein1	Acts as a tumor suppressor and participates in ATZ signaling pathway
ENSG0000074266	EED	0.01692107	Embryonic Ectoderm Development	Protein mediates repression of gene activities through histone deacetylation. Polycomb group such EzH2
ENSG00000168758	SEMA4C	0.00496868	Semaphorin 4C	Plays an important role in cell to cell signaling (promotes tumor growth and progression) maintaining tumor cell self-renewal
ENSG00000178562	CD28	0.039861847	CD 28 Molecular	Is one of the proteins that is expressed on T-cells that provide co-stimulatory signal required for T-cell activation and survival
ENSG00000132681	ATP1A4	0.043100268	ATPase Na+/K+ Transporting subfamily a 4	The protein encoded by this gene belongs to the family of P-type cation transport ATPases, and to the subfamily of Na+/K+ -ATPases. Na+/K+ -ATPase is an integral membrane protein responsible for establishing and maintaining the electrochemical gradients of Na and K ions across the plasma membrane.

**Table 2:** Raw data of proteomic detection and signal intensity generated via Label-Based Human Antibody Array L-2000 Glass Slide Kit from Ray Biotech. Antibodies were detected using fluorescence scanner and direct biotin labeling.. This data represents the top ten proteins with the greatest difference of expression measured in fold change downregulation (green color) of M0 vs M2 macrophages

Signal Intensity Fold Change of Differentiated Expressed Proteins in TAME in Comparison non TAME (Top 10 Downregulated)				
	Target Protein	Gene ID	Gene Name	Fold Change
1	GOLPH2	GOLM1	Golgi membrane protein 1	0.450
2	SORD	SORD	Sorbitol dehydrogenase	0.468
3	PSMB6	PSMB6	Proteasome subunit beta type-6	0.229
4	FDPS	FDPS	Farnesyl pyrophosphate synthase	0.731
5	Amylin	IAPP	Islet amyloid polypeptide	0.760
6	MINPP1	MINPP1	Multiple inositol polyphosphate phosphatase 1	0.750
7	Thrombospondin-1	THBS1	Thrombospondin-1	0.320
8	PSMA3	PSMA3	Proteasome subunit alpha type-3	0.410
9	LIMS1	LIMS1	LIM and senescent cell antigen-like-containing domain protein 1	0.868
10	VNN1	VNN1	Pantetheinase	0.543

**Table 3:** Raw data of proteomic detection and signal intensity generated via Label-Based Human Antibody Array L-2000 Glass Slide Kit from Ray Biotech. Antibodies were detected using fluorescence scanner and direct biotin labeling. This data represents the top ten proteins with the greatest difference of expression measured in fold change upregulation (red color) of M0 vs M2 macrophages. CSRP1 has the greatest upregulated fold change of 2.33

Signal Intensity Fold Change of Differentiated Expressed Proteins in TAME in Comparison to nonTAME (Top 10 Upregulated)				
	Target Protein	Gene ID	Gene Name	Fold Change
1	ORP150	HYOU1	Hypoxia up-regulated protein 1	1.57
2	ALAD	ALAD	Delta-aminolevulinic acid dehydratase	1.46
3	CSRP1	CSRP1	Cysteine and glycine-rich protein 1	2.33
4	C1qB	C1QB	Complement C1q subcomponent subunit B	1.27
5	beta-1 Spectrin	SPTB	Spectrin beta chain, erythrocytic	1.19
6	DNER	DNER	Delta and Notch-like epidermal growth factor-related receptor	1.95
7	eIF4A1	EIF4A1	Eukaryotic initiation factor 4A-I	1.12
8	Col6A2	COL6A2	Collagen alpha-2 (VI) chain	1.15
9	PEPD	PEPD	Xaa-Pro dipeptidase	1.21
10	ADH	ADH1A	Alcoholdehydrogenase	1.29

## Data Availability

All data will be available from the corresponding author upon reasonable request.
