# Supplementary material for: An Investigation to Study Morphology, Nanomechanical Attributes, Transcriptomics, and Proteomics of Tumor Associated Macrophages Derived Extracellular Vesicles in the Tumor Microenvironment
Source: J Anal Oncol. Author manuscript; Available in PMC 2026 Sep 10. (PMC13557178; doi:10.30683/1927-7229.2026.15.04)
Supplement: Supplementary file 1 [file NIHMS2194549-supplement-Supplementary_Figure.docx]

An Investigation to Study Morphology, Nanomechanical Attributes, Transcriptomics, and Proteomics of Tumor Associated Macrophages Derived Extracellular Vesicles in the Tumor Microenvironment

Kulkarni TK^1^, Banerjee A^2^, Banerjee N^4^, Cuffee J^4^, Newell S^4^ , Armstrong E^4^ , DeloatchSN^4^, Jong Park J^3^, El-Hashash AH^4^, Bhattacharya S^1^ , Banerjee H^4^

Supplementary Figure 1.

**Figure S1. Representative surface topography images of macrophages derived exosomes under various conditions.** A, B) Height image and C, D) Peak force error image of exosomes from M0 macrophage. E, F) Height image and G, H) Peak force error image of exosomes from TAM**.**
